# The European Board of transplant medicine: evolution, present role and future directions toward harmonised transplant medicine training in Europe

**DOI:** 10.3389/ti.2026.16968

**Published:** 2026-07-21

**Authors:** Nuria Montero, Miha Arnol, Susanne Beckebaum, Isabelle Binet, Vito Cicinnati, David Cucchiari, Caroline Den Hoed, Antoine Durrbach, Speranta Iacob, Smaragdi Marinaki, Anna Paola Mitterhofer, Anna Mrzljak, Adam Remport, Pablo Ruiz, Susana Sampaio, Francesca Tinti, Marios Prikis

**Affiliations:** 1 Nephrology Department, Hospital Universitari de Bellvitge, Barcelona, Spain; 2 Institut d’Investigació Biomèdica de Bellvitge (IDIBELL), L’ Hospitalet de Llobregat, Barcelona, Spain; 3 Department of Nephrology, University Medical Centre Ljubljana, Ljubljana, Slovenia; 4 Faculty of Medicine, University of Ljubljana, Ljubljana, Slovenia; 5 Department of Gastroenterology, Hepatology and Transplant Medicine, University Hospital Essen, Essen, Germany; 6 Nephrology and Transplantation Medicine, Health Ostschweiz, Cantonal Hospital St. Gallen, St. Gallen, Switzerland; 7 Renal Transplant Unit, Hospital Clínic, Barcelona, Spain; 8 Universitat de Barcelona, Barcelona, Spain; 9 Department of Gastroenterology and Hepatology, Erasmus MC University Medical Center, Erasmus MC Transplant Institute, Rotterdam, Netherlands; 10 University Paris Saclay, INSERM UMR 1356, Nephrology Department, Hopital Henri Mondor, Assistance Publique Hopitaux de Paris, Créteil, France; 11 Carol Davila University of Medicine and Pharmacy, Bucharest, Romania; 12 Center for Digestive Diseases and Liver Transplant, Fundeni Clinical Institute, Bucharest, Romania; 13 Clinic of Nephrology and Renal Transplantation, Medical School of Athens, Laiko Hospital, Kapodistrian University of Athens (NKUA), Athens, Greece; 14 Department of System Medicine, University of Rome Tor Vergata, Nephrology and Dyalisis Unit University Hospital Policlinico Tor Vergata, Rome, Italy; 15 Department of Gastroenterology and Hepatology, Liver Transplant Centre, University Hospital Centre Zagreb, School of Medicine, University of Zagreb, Zagreb, Croatia; 16 Department of Surgery, Transplantation and Gastroenterology, Semmelweis University, Budapest, Hungary; 17 Liver Unit, Hospital Clínic Barcelona, Institut d’Investigacions Biomèdiques August Pi i Sunyer (IDIBAPS), Universitat de Barcelona, Barcelona, Spain; 18 Kidney Transplant Unit, Unidade Local de Saúde (ULS) S João, Oporto, Portugal; 19 Faculdade Medicina, Oporto University, Oporto, Portugal; 20 Department of Clinical and Molecular Medicina, Nephrology and Dialysis Unit University Hospital S. Andrea, Sapienza University of Rome, Rome, Italy; 21 Nephrology Department, University of Vermont and Larner College of Medicine, Burlington, VT, United States

**Keywords:** education and training, harmonization, hepatology, nephrology, transplant care

Over the past four decades, organ replacement has evolved into a cornerstone of modern medicine. Nowadays kidney and liver transplantation offer durable and life-saving therapy for patients with end-stage organ failure. The field is no longer defined solely by surgical innovation, it also encompasses organ preservation technologies (including machine perfusion), xenotransplantation, precision immunology, artificial organs and regenerative medicine. Transplant physicians, particularly transplant nephrologists and hepatologists given the higher transplant volumes in these specialties, are now deeply involved in the evaluation of transplant candidates, perioperative decision-making, and lifelong post-transplant care. These responsibilities require specific expertise that extends beyond general hepatology or nephrology training. Despite this clinical maturity, transplant hepatology and transplant nephrology remain inconsistently structured across Europe and are not formally recognised as subspecialties in most Union Européenne des Médecins Spécialistes (UEMS) member states. Training pathways, certification mechanisms, and competency standards are heterogeneous and not uniformly defined.

The aim of this article is to provide an update on the EBTM’s current activities, increase awareness of its role in transfusion medicine, and promote broader engagement across European countries.

UEMS is a professional organisation that represents medical specialists across Europe. Since its foundation in 1958, UEMS has worked to promote high standards in specialist medical training, education, and clinical practice. The Division of Transplantation of UEMS was established in 2007 to address these challenges in the field of transplantation medicine, following preparatory work by the Transplant Working Group of the UEMS Section of Surgery and the European Board of Surgery. In 2010, the European Board of Transplantation Medicine (EBTM) was formally constituted as a joint initiative of UEMS and the European Society for Organ Transplantation (ESOT) reflecting both regulatory and scientific perspectives. Its objective is not to replace national systems but to promote a harmonized European framework capable of ensuring high standards of professional training and patient care. From its inception, the EBTM was designed as a non-profit body with a clear mandate, to safeguard the highest standards of care in transplantation medicine by harmonising postgraduate specialist training across Europe. The Board is composed of transplantation medicine physicians that are national representatives from UEMS member states, together with a representative appointed by ESOT.

The EBTM does not license physicians and does not interfere with national certification processes. Rather, it provides a structured, comprehensive and harmonized European-level assessment of Transplant Medicine that complements national training. The EBTM’s collaboration with ESOT has the potential to further support the standardisation of specialist qualifications, facilitate cross-border recognition of medical specialists, and contribute to shared quality benchmarks in European healthcare. The overarching objective of these joint initiatives is to ensure the highest standards of transplantation care for patients across Europe. ESOT is a strategic academic partner of the EBTM, and this collaboration is already operational in several concrete ways, including its basis in existing agreements such as the Memorandum of Understanding (MoU), where applicable. ESOT has an appointed representative on the EBTM Board who participates in all Board meetings and also serves as an examiner during the certification examinations. In addition, the EBTM examinations are held in conjunction with the ESOT Congress, with ESOT providing logistical support, including the venue. Furthermore, both organisations are currently strengthening their educational collaboration through ESOT’s existing learning platform, Transplant Live, which already hosts relevant courses and modules aligned with EBTM content.

A central activity of the EBTM has been the development of a structured modular examination system [1, 2]. The examination currently consists of three modules: (a) Common pathway module, (b) Kidney transplantation module, and (c) Liver transplantation module. Future goal is the expansion to include examinations in other organs such as pancreas, heart and lung. The Common Trunk evaluates foundational knowledge applicable across organ systems, including transplant immunology, mechanisms of rejection, immunosuppressive pharmacology, infectious complications, organ allocation systems, and ethical and moral considerations. The organ-specific modules assess advanced clinical expertise in kidney or liver such as waiting list management, organ donation and allocation principles, perioperative management of organ recipients and management of both short and long-term medical complications such as infections, malignancies and allograft rejection and dysfunction as well as immunosuppressive protocols. The certification by UEMS is a 2-step process based on the evaluation of training and clinical practice of the applicant, and an oral examination conducted in English, enabling assessment of integrative clinical reasoning and ethical judgment through case-based discussion. All the information required to apply is available on the website of the UEMS (https://uemssurg.org/surgicalspecialties/transplant-medicine/ebsq-examinations). Successful candidates are awarded the Fellowship of the European Board of Transplant Medicine, a credential that is increasingly recognised within the European transplant community and, in this sense, has institutional, pan-European recognition through the UEMS structure. The practical advantages associated with the credential vary significantly depending on the national and institutional context. In some countries or institutions, it may be valued as a marker of additional expertise and may support career progression, academic recognition, or professional credibility, while in others its impact may be more limited or informal.

Transplant Medicine practice has significantly evolved in most European countries. Yet, training national standards and fellowship pathways differ substantially across european borders (Table 1). Comparative analyses of individual state systems demonstrate significant diversity in transplant medical training. In some, transplant exposure is structured, while in others it is not. Both transplant nephrology and transplant hepatology, although clinically established, are not formally recognised as a subspecialties in any surveyed systems [3]. An isolated exception seems to be Germany, with an officially recognised national certification pathway in Transplant Medicine.

**TABLE 1:** Overview of transplant nephrology and hepatology training across selected European countries.

Country	Parent specialty	Formal transplant subspecialty recognition	Training pathway (summary)	Transplant exposure	Perceived value of EBTM certification*
Cyprus	Nephrology	No	≥2 years internal medicine + 4 years nephrology	Mandatory (≥3 months in transplant centre)	Objective validation and harmonisation
	Gastroenterology/Hepatology	No	2 years internal medicine + 4 years gastroenterology	Integrated	Objective validation and harmonisation
Croatia	Nephrology	No	5-year nephrology residency	Integrated	Objective validation and harmonisation
	Gastroenterology/Hepatology	No	5-year gastroenterology residency	Integrated	Objective validation and harmonisation
France	Nephrology	No	5-year residency + 2-year clinical fellowship	Integrated within nephrology	Harmonisation and professional mobility
	Gastroenterology/Hepatology	No	5-year gastroenterology residency	Integrated	Objective validation and harmonisation
Germany**	Nephrology	**Yes**	Board certification in internal medicine + nephrology + ≥2 years transplant nephrology	Mandatory	Complementary to existing national certification
	Gastroenterology/Hepatology	**Yes**	Board certification in internal medicine + gastroenterology + ≥2 years transplant hepatology	Mandatory	Complementary to existing national certification
Greece	Nephrology	No	2 years internal medicine + 4 years nephrology	Mandatory (≥3 months in transplant centre)	Fills training gap; supports hiring in transplant centres
	Gastroenterology/Hepatology	No	2 years internal medicine + 4 years gastroenterology	Integrated	Fills training gap; european-level credential
Hungary	Nephrology	No	5-year nephrology or internal medicine + 2 years nephrology	Variable	Standardisation and capacity building
	Gastroenterology/Hepatology	No	5-year gastroenterology residency	Integrated	Fills educational gap; capacity building
Italy	Nephrology	No	National competitive residency; no transplant certification	Variable	Fills training gap; harmonisation
	Gastroenterology/Hepatology	No	4-year gastroenterology residency	Variable	Fills educational gap
Netherlands	Nephrology	No	4 years internal medicine + 2 years nephrology (transplant mandatory)	Mandatory	Fills educational gap
	Gastroenterology/Hepatology	No	6-year combined GI–Hepatology training	Optional hepatology registration	Standardised validation
Portugal	Nephrology	No	5-year residency including transplantation	Integrated	Certification and quality standardisation
	Gastroenterology/Hepatology	No	5–6 years GI/Internal medicine + additional liver training	Variable	Certification and quality standardisation
Romania	Nephrology	No	5-year nephrology residency; transplant via accredited centres	Centre-dependent	European benchmark; supports professional mobility
	Gastroenterology/Hepatology	No	5-year gastroenterology residency	Integrated	European benchmark
Slovenia	Nephrology	No	6-year regulated nephrology residency; national exam	Integrated	Objective validation and harmonisation
	Gastroenterology/Hepatology	No	6-year regulated gastroenterology residency; national exam	Integrated	Objective validation and harmonisation
Spain	Nephrology	No	4-year residency (MIR); transplant exposure mandatory	Mandatory	European-level credential
	Gastroenterology/Hepatology	No	4-year GI/Hepatology residency	Variable	Unifies training criteria
Switzerland	Nephrology	No	2–3 years internal medicine + 3–4 years nephrology; national + UEMS exams	Integrated	European standards embedded
	Gastroenterology/Hepatology	No	3 years internal medicine + 3 years gastroenterology + hepatology subspecialty year	Integrated	European standards embedded

* This measure reflects stakeholders’ perceptions of the value and professional impact of the EBTM, certification across different clinical and academic environments.

** Germany is the only surveyed country with formally recognised national certification in Transplant Medicine.

Core duties across all countries encompass pre-transplant candidate evaluation, perioperative management, and lifelong post-transplant care.

Abbreviations: EBTM, european board of transplantation medicine; GI, gastroenterology; MIR, Médico Interno Residente; UEMS, Union Européenne des Médecins Spécialistes; yrs, years.

The EBTM framework defines minimum requirements regarding duration of structured transplant training, documented clinical exposure, participation in multidisciplinary teams, core knowledge domains, and ethical and professional competencies (Table 2). These standards promote consistency while respecting national autonomy. Applicants are evaluated through a robust review process to determine eligibility for the examination. We have a review committee in place: the President appoints three blinded reviewers, ensuring they are not aware of each other’s involvement. In the event of discrepancies in their evaluations, the review committee convenes to discuss and reach a consensus, and the matter is then escalated to the full Board when necessary. Additionally, we contact referees via telephone or email to gather further information. Eligibility for EBTM certification requires both clinical practice and scientific development aspects, such as documented participation in congresses, courses, and educational activities. Following a thorough evaluation of the relevant competencies, the eligible examinees can participate in the examination for the obligatory common pathway module and then subsequently, depending on their training either the transplant hepatology or nephrology modules.

**TABLE 2:** EBTM eligibility requirements for certification.

Domain	Requirements
Training duration and background	• Board certification in internal medicine or paediatrics and a recognised subspecialty (e.g., Nephrology, gastroenterology/Hepatology, cardiology, pulmonology) • Minimum 2 years of continuous transplant medicine training (up to 4 years if part-time) • Training must be completed or validated in a UEMS member country
Clinical competencies	• Documented experience in pre-transplant evaluation, inpatient management, and outpatient follow-up • Evidence of supervised and independently performed specialised procedures • Attestation by a senior transplant physician or head of department confirming patient caseload and procedural exposure
Education and academic activity	• Attendance at recognised transplant courses (including ESOT educational programmes), national and international transplant congresses, and CME-accredited symposia • Mandatory: Participation in a minimum of two national/international transplant meetings with first or last authorship (oral or poster presentation)
Non-technical skills	• Proficiency in English (required for the oral examination)

Abbreviations: CME, continuing medical education; ESOT, european society for organ transplantation; UEMS, Union Européenne des Médecins Spécialistes.

The EBTM examination is currently held every 2 years within the framework of the ESOT Congress. The next examination will be held in September 2027. It is an in-person, oral, case-based, and competency-driven assessment that evaluates integrative reasoning rather than simple factual recall. Its governance is transparent, with clearly defined eligibility criteria and independent panels of examiners.

The EBTM provides structured curriculum standards, defined eligibility criteria, and established examination processes that could support subspecialty recognition in Transplant Medicine [4], should national and European regulators decide to pursue this pathway. Looking ahead, EBTM’s strong efforts to build partnerships with ESOT and the sections of EKITA (The European Kidney Transplant Association) and ELITA (The European Liver and Intestine Transplant Association) as well as other UEMS specialty sections, will strengthen its role and mission, assisting in progressively building a broader, harmonized European transplant medicine training and certification framework. Joint efforts could focus on aligning curricula with evolving clinical needs, integrating EBTM-related educational activities within ESOT’s portfolio, and promoting shared competency frameworks for all organ transplantation disciplines. By leveraging ESOT’s educational platforms and the EBTM’s examination standards, such initiatives could contribute to more harmonized competencies and facilitate cross-border recognition of expertise. Consequently, EBTM’s is committed to expanding its certification beyond nephrology and hepatology, enhancing the recognition, visibility, and adoption across Europe, and pursuing formal recognition of subspecialties such as transplant nephrology and hepatology. Despite ongoing efforts to promote engagement, participation in the EBTM framework remains incomplete across European countries, underscoring the need for continued outreach and expansion to achieve broader European integration of transplant medicine training standards. Extending the certification to additional Transplant Medicine disciplines such as heart, lung, and pancreas transplantation would further harmonise competencies across the full spectrum of transplant care and better reflect its multidisciplinary nature. To strengthen awareness and adoption, key strategies include closer integration with ESOT educational activities, increased engagement with national societies and training programmes, focused outreach initiatives, and greater promotion of the certification as a tool for facilitating professional mobility and standardised expertise across Europe. Eligibility for certification by the European Transplant Medicine Board is open to European clinicians, as well as to non-European clinicians who have completed and validated a period of clinical practice in one of the member UEMS countries.

In conclusion, the EBTM represents meaningful and maturing step towards the harmonization of Transplant medicine training and certification across Europe. By establishing structured curricula, defined competency framework, and rigorous examination standards, it offers a valuable complement to existing national systems and contributes to the broader recognition of transplant nephrology and hepatology as distinct clinical disciplines. Continued efforts to expand geographic participation, extend certification to additional transplant disciplines, and strengthen collaboration with ESOT, UEMS sections and national societies will be vital in further consolidating its role. Together, these initiatives reflect a shared commitment to promoting high standards of transplant medicine training and, ultimately, to improving the quality of care for transplant patients across Europe.

## Data Availability

The original contributions presented in the study are included in the article/supplementary material, further inquiries can be directed to the corresponding authors.
