# Correction: The European Board of transplant medicine: evolution, present role and future directions toward harmonised transplant medicine training in Europe

**DOI:** 10.3389/ti.2026.17461

**Published:** 2026-08-28

**Authors:** Nuria Montero, Miha Arnol, Susanne Beckebaum, Isabelle Binet, Vito Cicinnati, David Cucchiari, Caroline Den Hoed, Antoine Durrbach, Speranta Iacob, Smaragdi Marinaki, Anna Paola Mitterhofer, Anna Mrzljak, Adam Remport, Pablo Ruiz, Susana Sampaio, Francesca Tinti, Marios Prikis

**Affiliations:** 1 Nephrology Department, Hospital Universitari de Bellvitge, Barcelona, Spain; 2 Institut d’Investigació Biomèdica de Bellvitge (IDIBELL), L’ Hospitalet de Llobregat, Barcelona, Spain; 3 Department of Nephrology, University Medical Centre Ljubljana, Ljubljana, Slovenia; 4 Faculty of Medicine, University of Ljubljana, Ljubljana, Slovenia; 5 Department of Gastroenterology, Hepatology and Transplant Medicine, University Hospital Essen, Essen, Germany; 6 Nephrology and Transplantation Medicine, Health Ostschweiz, Cantonal Hospital St. Gallen, St. Gallen, Switzerland; 7 Renal Transplant Unit, Hospital Clínic, Barcelona, Spain; 8 Universitat de Barcelona, Barcelona, Spain; 9 Department of Gastroenterology and Hepatology, Erasmus MC University Medical Center, Erasmus MC Transplant Institute, Rotterdam, Netherlands; 10 University Paris Saclay, INSERM UMR 1356, Nephrology Department, Hopital Henri Mondor, Assistance Publique Hopitaux de Paris, Créteil, France; 11 Carol Davila University of Medicine and Pharmacy, Bucharest, Romania; 12 Center for Digestive Diseases and Liver Transplant, Fundeni Clinical Institute, Bucharest, Romania; 13 Clinic of Nephrology and Renal Transplantation, Medical School of Athens, Laiko Hospital, Kapodistrian University of Athens (NKUA), Athens, Greece; 14 Department of System Medicine, University of Rome Tor Vergata, Nephrology and Dyalisis Unit University Hospital Policlinico Tor Vergata, Rome, Italy; 15 Department of Gastroenterology and Hepatology, Liver Transplant Centre, University Hospital Centre Zagreb, School of Medicine, University of Zagreb, Zagreb, Croatia; 16 Department of Surgery, Transplantation and Gastroenterology, Semmelweis University, Budapest, Hungary; 17 Liver Unit, Hospital Clínic Barcelona, Institut d’Investigacions Biomèdiques August Pi i Sunyer (IDIBAPS), Universitat de Barcelona, Barcelona, Spain; 18 Kidney Transplant Unit, Unidade Local de Saúde (ULS) S João, Oporto, Portugal; 19 Faculdade Medicina, Oporto University, Oporto, Portugal; 20 Department of Clinical and Molecular Medicina, Nephrology and Dialysis Unit University Hospital S. Andrea, Sapienza University of Rome, Rome, Italy; 21 Nephrology Department, University of Vermont and Larner College of Medicine, Burlington, VT, United States

**Keywords:** education and training, harmonization, hepatology, nephrology, transplant care

Table one and Table two were erroneously omitted from this article. The tables appear below.

**TABLE 1:** Overview of transplant nephrology and hepatology training across selected European countries.

Country	Parent specialty	Formal transplant subspecialty recognition	Training pathway (summary)	Transplant exposure	Perceived value of EBTM certification*
Cyprus	Nephrology	No	≥2 years internal medicine + 4 years nephrology	Mandatory (≥3 months in transplant centre)	Objective validation and harmonisation
	Gastroenterology/Hepatology	No	2 years internal medicine + 4 years gastroenterology	Integrated	Objective validation and harmonisation
Croatia	Nephrology	No	5-year nephrology residency	Integrated	Objective validation and harmonisation
	Gastroenterology/Hepatology	No	5-year gastroenterology residency	Integrated	Objective validation and harmonisation
France	Nephrology	No	5-year residency + 2-year clinical fellowship	Integrated within nephrology	Harmonisation and professional mobility
	Gastroenterology/Hepatology	No	5-year gastroenterology residency	Integrated	Objective validation and harmonisation
Germany**	Nephrology	**Yes**	Board certification in internal medicine + nephrology + ≥2 years transplant nephrology	Mandatory	Complementary to existing national certification
	Gastroenterology/Hepatology	**Yes**	Board certification in internal medicine + gastroenterology + ≥2 years transplant hepatology	Mandatory	Complementary to existing national certification
Greece	Nephrology	No	2 years internal medicine + 4 years nephrology	Mandatory (≥3 months in transplant centre)	Fills training gap; supports hiring in transplant centres
	Gastroenterology/Hepatology	No	2 years internal medicine + 4 years gastroenterology	Integrated	Fills training gap; european-level credential
Hungary	Nephrology	No	5-year nephrology or internal medicine + 2 years nephrology	Variable	Standardisation and capacity building
	Gastroenterology/Hepatology	No	5-year gastroenterology residency	Integrated	Fills educational gap; capacity building
Italy	Nephrology	No	National competitive residency; no transplant certification	Variable	Fills training gap; harmonisation
	Gastroenterology/Hepatology	No	4-year gastroenterology residency	Variable	Fills educational gap
Netherlands	Nephrology	No	4 years internal medicine + 2 years nephrology (transplant mandatory)	Mandatory	Fills educational gap
	Gastroenterology/Hepatology	No	6-year combined GI–Hepatology training	Optional hepatology registration	Standardised validation
Portugal	Nephrology	No	5-year residency including transplantation	Integrated	Certification and quality standardisation
	Gastroenterology/Hepatology	No	5–6 years GI/Internal medicine + additional liver training	Variable	Certification and quality standardisation
Romania	Nephrology	No	5-year nephrology residency; transplant via accredited centres	Centre-dependent	European benchmark; supports professional mobility
	Gastroenterology/Hepatology	No	5-year gastroenterology residency	Integrated	European benchmark
Slovenia	Nephrology	No	6-year regulated nephrology residency; national exam	Integrated	Objective validation and harmonisation
	Gastroenterology/Hepatology	No	6-year regulated gastroenterology residency; national exam	Integrated	Objective validation and harmonisation
Spain	Nephrology	No	4-year residency (MIR); transplant exposure mandatory	Mandatory	European-level credential
	Gastroenterology/Hepatology	No	4-year GI/Hepatology residency	Variable	Unifies training criteria
Switzerland	Nephrology	No	2–3 years internal medicine + 3–4 years nephrology; national + UEMS exams	Integrated	European standards embedded
	Gastroenterology/Hepatology	No	3 years internal medicine + 3 years gastroenterology + hepatology subspecialty year	Integrated	European standards embedded

* This measure reflects stakeholders’ perceptions of the value and professional impact of the EBTM, certification across different clinical and academic environments.

** Germany is the only surveyed country with formally recognised national certification in Transplant Medicine.

Core duties across all countries encompass pre-transplant candidate evaluation, perioperative management, and lifelong post-transplant care.

Abbreviations: EBTM, european board of transplantation medicine; GI, gastroenterology; MIR, Médico Interno Residente; UEMS, Union Européenne des Médecins Spécialistes; yrs, years.

**TABLE 2:** EBTM eligibility requirements for certification.

Domain	Requirements
Training duration and background	• Board certification in internal medicine or paediatrics and a recognised subspecialty (e.g., Nephrology, gastroenterology/Hepatology, cardiology, pulmonology) • Minimum 2 years of continuous transplant medicine training (up to 4 years if part-time) • Training must be completed or validated in a UEMS member country
Clinical competencies	• Documented experience in pre-transplant evaluation, inpatient management, and outpatient follow-up • Evidence of supervised and independently performed specialised procedures • Attestation by a senior transplant physician or head of department confirming patient caseload and procedural exposure
Education and academic activity	• Attendance at recognised transplant courses (including ESOT educational programmes), national and international transplant congresses, and CME-accredited symposia • Mandatory: Participation in a minimum of two national/international transplant meetings with first or last authorship (oral or poster presentation)
Non-technical skills	• Proficiency in English (required for the oral examination)

Abbreviations: CME, continuing medical education; ESOT, european society for organ transplantation; UEMS, Union Européenne des Médecins Spécialistes.

The original version of this article has been updated.

